# Diagnostic value of neutrophil CD64, procalcitonin, and interleukin-6 in sepsis: a meta-analysis

**DOI:** 10.1186/s12879-021-06064-0

**Published:** 2021-04-26

**Authors:** Shan Cong, Tiangang Ma, Xin Di, Chang Tian, Min Zhao, Ke Wang

**Affiliations:** Department of Respiratory Medicine, The Second Hospital of Jilin University, 218 Ziqiang Street, Nanguan District, Changchun, 130041 Jilin Province China

**Keywords:** Sepsis, Neutrophil CD64, Procalcitonin, Interleukin-6, Meta-analysis

## Abstract

**Background:**

The aim of the study was to conduct a meta-analysis to evaluate the accuracy of neutrophil CD64, procalcitonin (PCT), and interleukin-6 (IL-6) as markers for the diagnosis of sepsis in adult patients.

**Methods:**

Various databases were searched to collect published studies on the diagnosis of sepsis in adult patients using neutrophil CD64, PCT, and IL-6 levels. Utilizing the Stata SE 15.0 software, forest plots and the area under the summary receiver operating characteristic curves were drawn. The pooled sensitivity, specificity, positive likelihood ratio, negative likelihood ratio, diagnostic odds ratio, and area under the curve (AUC) were calculated.

**Results:**

Fifty-four articles were included in the study. The pooled sensitivity, specificity, and AUC of neutrophil CD64 for the diagnosis of sepsis were 0.88 (95% confidence interval [CI], 0.81–0.92), 0.88 (95% CI, 0.83–0.91), and 0.94 (95% CI, 0.91–0.96), respectively. The pooled sensitivity, specificity, and AUC of PCT for the diagnosis of sepsis were 0.82 (95% CI, 0.78–0.85), 0.78 (95% CI, 0.74–0.82), and 0.87 (95% CI, 0.83–0.89), respectively. Subgroup analysis showed that the AUC for PCT diagnosis of intensive care unit (ICU) sepsis was 0.86 (95% CI, 0.83–0.89) and the AUC for PCT diagnosis of non-ICU sepsis was 0.82 (95% CI, 0.78–0.85). The pooled sensitivity, specificity, and AUC of IL-6 for the diagnosis of sepsis were 0.72 (95% CI, 0.65–0.78), 0.70 (95% CI, 0.62–0.76), and 0.77 (95% CI, 0.73–0.80), respectively.

**Conclusions:**

Of the three biomarkers studied, neutrophil CD64 showed the highest diagnostic value for sepsis, followed by PCT, and IL-6. On the other hand, PCT showed a better diagnostic potential for the diagnosis of sepsis in patients with severe conditions compared with that in patients with non-severe conditions.

## Background

In recent years, the incidence and mortality of sepsis have increased significantly due to the increase of drug-resistant bacteria, the widespread use of antibiotics, and the aging of the population. The latest epidemiological study, including septicemia cases in 195 countries around the world, showed that in 2017 there were 48.9 million sepsis patients and 11 million deaths from sepsis worldwide, which was equivalent to 19.7% of total deaths throughout the year [1]. In 2016, the Society of Critical Care Medicine (SCCM) and the European Society of Intensive Care Medicine (ESICM) jointly issued the definition of Sepsis 3.0 as the life-threatening organ dysfunction caused by dysregulation of the host’s response to infection [2]. At the same time, the diagnostic criteria for sepsis were proposed. For patients with ICU infection or suspected infection, sepsis is diagnosed when the sequential organ failure assessment (SOFA) score is ≥2 [3]. However, considering the limitations of the diagnostic criteria and the lack of clinically relevant data in many patients, a simplified method was proposed, named “quick SOFA”, (also known as “qSOFA”), that includes a systolic blood pressure ≤ 100 mmHg, a respiratory frequency ≥ 22 times/min, or change of consciousness. When there are two or more score exceptions, this can be considered a high-risk sepsis population [4]. However, Williams et al. [5] found that although qSOFA score was highly specific, its sensitivity was poor, which might not be suitable for early diagnosis of sepsis. Although blood culture is an important tool for sepsis diagnosis that identifies pathogenic bacteria and allows antibiotic susceptibility testing, it is a time-consuming protocol and has a high false-negative rate, especially after antibiotic use [6]. Therefore, the blood culture alone is not enough to assist clinicians to make accurate early diagnosis in patients with sepsis. According to statistics, if sepsis patients can be correctly diagnosed and treated within 1 h of infection, their survival rate will reach more than 80%, whereas if patients are diagnosed and treated after 6 h of infection, their survival rate drops to 30% [7]. Therefore, it is crucial to find a biomarker for the early diagnosis of sepsis.

Neutrophil CD64 is a high-affinity receptor for the Fc portion of IgG. Neutrophil CD64 is a member of the immunoglobulin superfamily and is mainly found on the surface of antigen-presenting cells, such as monocytes, macrophages, and dendritic cells. When the body is infected, or a large number of bacterial endotoxins are present, neutrophils are exposed to lipopolysaccharides (LPS), complement system molecules, IL-8, IL-12, IFN-γ, TNF-α, granulocyte colony-stimulating factor, and other cytokines. Such molecules stimulate the expression of CD64 and it remains stable for a certain period of time [8]. Although neutrophil CD64 expression is low on resting neutrophils, once activated by stimulating factors its expression increases rapidly up to10-fold, reaching a peak within 4 to 6 h. Basal expression is restored 7 days after the stimulation disappears [9]. Neutrophil CD64 is relatively stable in blood samples studied in vitro and is easily detected by flow cytometry. The stable characteristics of neutrophil CD64 make it suitable as a diagnostic indicator.

Biomarkers procalcitonin (PCT) and interleukin-6 (IL-6) have been widely used in the diagnosis and identification of infections. Under normal physiological conditions, PCT is produced almost exclusively in thyroid C cells. Induced by the stimulation of glucocorticoids, calcitonin gene-related peptide, glucagon, gastrin, or β-adrenergic signaling, PCT is converted into calcitonin before entering the circulatory system. Healthy individuals usually show very low levels of serum PCT (< 0.02 ng/mL). PCT is a very stable protein in vitro and in vivo, with a half-life of about 20–24 h [10, 11]. Patients with infections can produce PCT through an alternative pathway in non-thyroid tissue. There are two main alternative pathways: the direct pathway, induced by LPS or other toxic microbial metabolites, and the indirect pathway, induced by several inflammatory mediators such as IL-6 and TNF-α [12]. Due to the lack of pathways to convert PCT to calcitonin, PCT enters the circulatory system and its levels can rapidly increase more than 400-fold (> 4.0 ng/mL) compared to basal levels [13].

IL-6 is an important pro-inflammatory factor in the initial stage of inflammation. It induces multiple cells to synthesize and secrete acute phase proteins, promotes the production and activation of neutrophils during infection, promotes the proliferation and differentiation of B cells, produces immunity globulins, and promotes T cell proliferation and differentiation [14]. The levels of IL-6 in healthy people are extremely low, generally not exceeding 7 pg/mL, whereas the levels of IL-6 in the serum of sepsis patients increases rapidly in the early stage of infection, and can reach a peak within 2 h [15].

The aim of our study was to integrate the results of clinical studies to compare the diagnostic accuracy of neutrophil CD64, PCT, and IL-6 for sepsis in adult patients by meta-analysis.

## Materials and methods

### Study selection

The articles were manually retrieved from PubMed, Web of Science, Medline, The Cochrane Library, Wan Fang, China Biology Medicine, China National Knowledge Infrastructure, and VIP databases, by searching all publications from the earliest entries to December 2018. Languages were English and Chinese. Firstly, the studies were chosen based on the following subject terms: sepsis, neutrophil CD64, procalcitonin, Interleukin-6, and diagnosis. Then, a relevant-free terms search was carried out, and finally, the two search strategies were combined. Additionally, the references cited in the retrieved articles were also manually retrieved as supplements. Endnote version X7.8 was used for reference management. Two researchers carried out the same search independently, and in case of disagreement, a third researcher was involved to discuss the results and reach an agreement.

### Inclusion and exclusion criteria

#### Inclusion criteria

1. Studies focused on the diagnostic value of neutrophil CD64, PCT, and IL-6 for sepsis; 2. The observation group included adult sepsis patients, aged ≥18 years, whereas the control group included patients or healthy people assessed during the same period; 3.The diagnostic criteria included the clinical diagnostic and or blood culture. The clinical diagnostic criteria were Sepsis 1.0, Sepsis 2.0, and Sepsis 3.0; 4. Prospective or retrospective studies; 5. True positive (TP), false positive (FP), true negative (TN), or false negative (FN) results for neutrophil CD64, PCT, and IL-6 in the diagnosis of sepsis could be obtained directly or calculated from the data.

#### Exclusion criteria

1. Abstracts, conference reports, summaries, and comments; 2. TP, FP, TN, and FN cannot be obtained according to the reported data; 3. Repeated research subjects.

### Quality assessment

We used the diagnostic test system evaluation tool Quality Assessment for Diagnostic Accuracy Studies version 2 (QUADAS-2) from the Review Manager 5.3 software to assess the quality of all included articles. The QUADAS-2 scale includes four parts: case selection, trial to be evaluated, gold standard, and case process and progress.

### Data extraction

The research data extraction was independently completed by two researchers. If the extraction results of the two were inconsistent, the third researcher and the first two jointly studied and decided. The data extraction information included the first author, publication date, country, study design, diagnostic criteria, clinical setting, sample size, average age, test method, TP, FP, FN, TN, sensitivity, and specificity.

### Statistical analysis

This study was a diagnostic meta-analysis. The heterogeneity of the included articles was determined to select the appropriate statistical model to help reduce errors during data merging. The heterogeneity between the included studies was evaluated by calculating the chi-square test value and the I^2^ statistics. If the I^2^ ≤ 50%, *P* ≥ 0.05, the heterogeneity of the included studies was deemed small, and the fixed effect model was used to merge the statistical data. If the I^2^ > 50%, *P* < 0.05, the heterogeneity was significant, and data were merged by the random effect model. The indexes included sensitivity, specificity, positive likelihood ratio (PLR), negative likelihood ratio (NLR), and diagnostic odds ratio (DOR). Additionally, a summary receiver operating characteristic (SROC) curve was drawn to calculate the area under the curve (AUC). The closer the AUC value was to 1, the higher the clinical diagnostic efficacy of this index was. The Deeks’ test was used to assess publication bias in the included articles. We used meta-regression, sensitivity analysis, and subgroup analysis to explore the sources of heterogeneity. We used Fagan’s nomogram to evaluate the post-test probabilities of the three studied biomarkers in sepsis. MetaDisc 1.4 software and STATA 12.0 were used for data analysis.

## Results

### Literature search

**Fig. 1:**
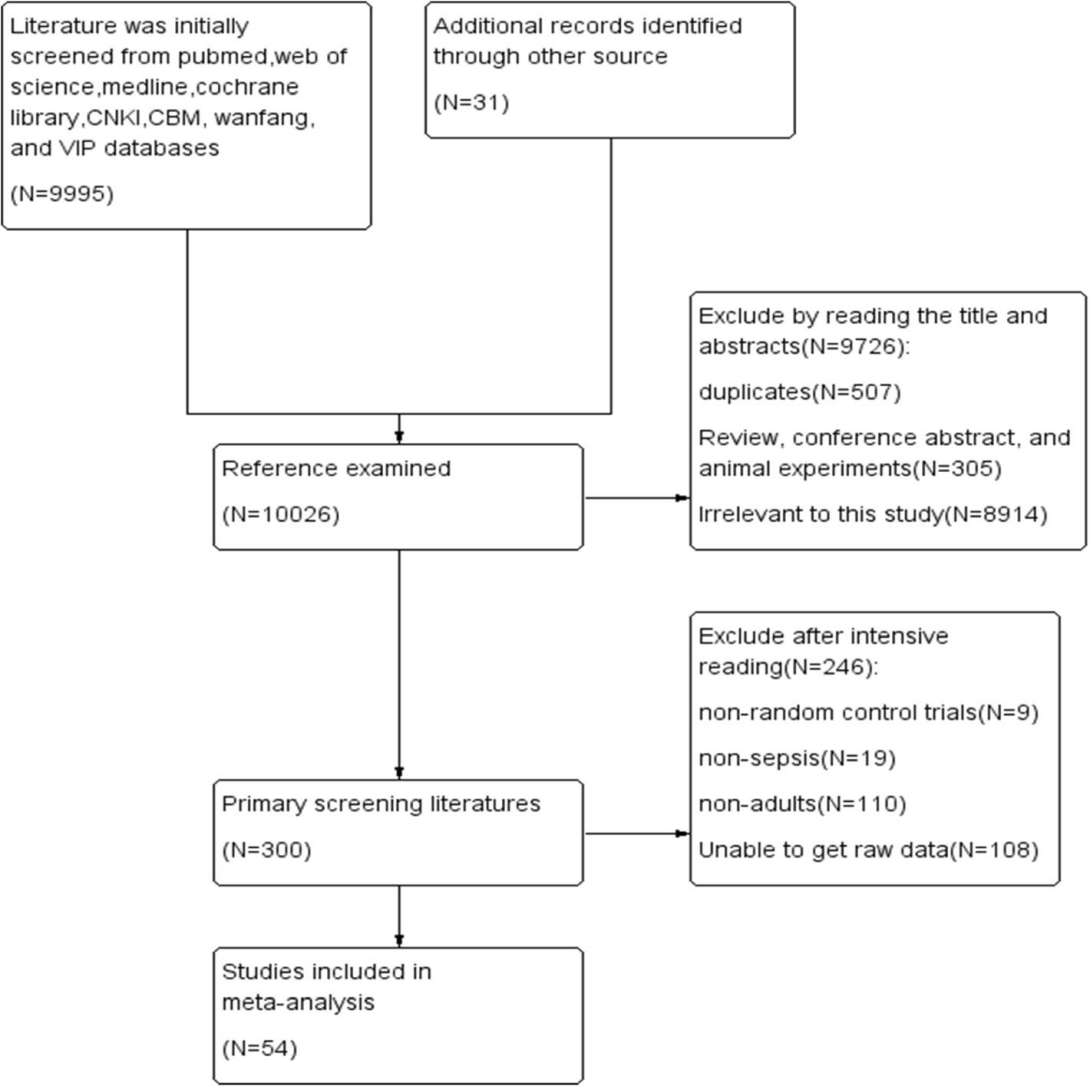
PRISMA flow diagram of the search strategy and study selection process

In all, 10,026 articles in Chinese and English were retrieved through the preliminary screening of the databases. After reading the titles and abstracts, 300 articles were selected. Intensive reading was performed following strictly the inclusion and exclusion criteria. After the screening, a set of 54 articles were included in the study (Fig. 1).

### Study characteristics

In all, 9842 participants were finally enrolled in this meta-analysis, with a sepsis prevalence of 54.8%. It included 20 studies related to neutrophil CD64, 39 studies related to PCT, and 15 studies related to IL-6. We found 37 articles that reported the average age of the study subjects, ranging from 42.0 to 92.6 years. Four papers focused on patients with specific sepsis, such as patients with acute abdominal sepsis [16], ventilator-associated pneumonia [17], and postoperative sepsis [18, 19]. Two articles addressed elderly patients with sepsis (aged > 65 [20] and > 85 years [21]), whereas another study [22] included patients aged ≤65 and > 65 years. Two studies [23, 24] reported separately cases of positive and negative blood cultures. One paper [25] included a study conducted in the medical ICU and surgical ICU patients. The detailed baseline characteristics of the included studies are summarized in Table 1.

**Table 1:** Baseline characteristics of included studies in the meta analysis

first author	publication time	country	study design	diagnostic criteria	clinical setting	biomarker	sample size	TP	FP	FN	TN	SEN (%)	SPE (%)	average age	test method
Anand [23]a	2015	India	PS	culture+	ICU	PCT	118	68	6	4	40	94.4	87	49.3	IF
						IL-6	118	46	5	26	41	63.9	89	49.3	ECLI
Anand [23] b	2015	India	PS	clinical,culture-	ICU	PCT	136	83	13	7	33	92.2	72	52.1	IF
						IL-6	136	42	12	48	34	47	73	52.1	ECLI
Bauer [26]	2016	America	PS	clinical	ICU	CD64	196	84	20	26	66	76.4	76.7		FCM
						PCT	216	88	25	32	71	73.1	74.2		IF
Cardelli [27]	2008	Italy	PS	clinical,culture+	ICU	CD64	112	50	5	2	55	96	91.7	63	FCM
						PCT	112	49	27	3	33	94	70	63	IF
Castelli [28]	2004	Italy	PS	clinical,culture+	ICU	PCT	49	21	2	13	13	61.7	86.7		IF
Cheval [29]	2000	France	PS	clinical	ICU	PCT	60	28	5	4	23	87.5	82.1	56.3	IF
Clec’h [25] a	2006	France	PS	clinical	SICU	PCT	67	28	9	3	27	91.7	74.2	63	IF
Clec’h [25] b	2006	France	PS	clinical	MICU	PCT	76	29	2	7	38	80.6	95	60.1	IF
Davis [30]	2006	America	RS	clinical,culture+	ED	CD64	100	33	18	5	44	86.8	71		FCM
Dimoula [31]	2014	Belgium	PS	clinical	ICU	CD64	468	92	47	11	318	89.3	87.1	58.7	FCM
Du [32]	2003	China	PS	clinical	ICU	PCT	51	16	8	4	23	80	74.2	64.7	IF
						IL-6	51	17	8	3	23	85	74	64.7	EIA
Feng [33]	2012	China	PS	clinical	ICU	PCT	132	69	12	33	18	67.6	60		ELISA
Gaini [34]	2006	Denmark	PS	clinical	GW	PCT	93	56	9	18	10	75.7	52.6	63	IF
						IL-6	93	60	4	14	15	81.1	78.9	63	ECLI
Gamez-Diaz [35]	2011	Colombia	PS	clinical	ED	CD64	610	266	73	138	133	65.8	65		Leuko64 kit
Gerrits [36]	2013	Netherla-nds	PS	clinical	ICU	CD64	44	25	1	0	18	100	94.7	71.8	Leuko64 kit
Gibot [37]	2012	France	PS	clinical	ICU	CD64	300	130	7	24	139	84.4	95.2	61.5	FCM
						PCT	300	128	30	26	124	83.1	84.9	61.5	ECLI
Gros [38]	2012	France	PS	clinical, culture+	MICU	CD64	293	93	16	55	129	62.8	89	59.5	Leuko64 kit
Gupta [24] a	2018	India	PS	culture+		PCT	242	193	5	3	41	98.5	89.1		ECLI
Gupta [24] b	2018	India	PS	clinical,culture-		PCT	109	55	10	8	36	87.3	78.3		ECLI
Harbarth [39]	2001	Switzerla-nd	PS	clinical	ICU	PCT	78	58	4	2	14	97	78		ECLI
						IL-6	78	40	5	20	13	67	72		ECLI
Hausfater [40]	2002	France	PS	clinical	ED	PCT	195	42	15	26	112	61.8	88.2	47	IFA
Hsu [41]	2011	China	PS	clinical, culture+	RICU	CD64	66	49	1	6	10	89	90.9	68.3	FCM
						PCT	66	31	0	24	11	56.4	100	68.3	IF
Ivancević [16]	2008	Serbia	PS	clinical,culture+	ED	PCT	98	42	15	16	25	72.4	62.5	54.7	IF
Jämsä [42]	2015	Finland	PS	clinical	ICU	CD64	42	27	1	0	14	100	93	64.4	FCM
Jekarl [43]	2012	Korea	PS	clinical	ED	PCT	177	58	13	20	86	74.4	86.7	51.5	ECLI
						IL-6	177	51	17	27	82	65.4	82.9	51.5	ECLI
Kofoed [44]	2007	Denmark	PS	clinical,culture+	ED/GW	PCT	151	77	23	19	32	80.2	58.2		ECLI
Latour-Pérez [45]	2010	Spain	PS	clinical	ICU	PCT	114	53	5	19	37	73.6	88.1		IF
Lewis [46]	2015	UK	RS	clinical, culture+	ICU	CD64	153	43	12	40	58	51.8	82.6		FCM
Mat-Nor [47]	2016	Malaysia	PS	clinical	ICU	PCT	239	93	20	71	55	57	73	47	IF
						IL-6	239	82	26	82	49	50	65	47	EI
Meynaar [48]	2011	Netherlands	PS	clinical,culture+	ICU	PCT	76	31	9	1	35	97	80		IF
						IL-6	76	29	26	3	18	91	41		ECLI
Mokart [18]	2005	France	PS	clinical	ICU	PCT	50	13	10	3	24	81	72		ECLI
						IL-6	50	14	14	2	20	87.5	58.8		EIA
Muller [49]	2000	Switzerla-nd	PS	clinical	MICU	PCT	101	53	3	6	39	89.8	92.9		IFA
						IL-6	101	38	9	21	33	64.4	78.6		EIA
Muzlovic [17]	2016	Slovenia	PS	clinical,culture+	ICU	CD64	32	25	1	0	6	100	85.7	61.8	Leuko64 kit
						PCT	32	21	0	4	7	81.8	100	61.8	IF
Papadimitriou [50]	2015	Greece	PS	clinical,culture+	ICU	CD64	66	24	3	5	34	83	92		FCM
Righi [51]	2014	Italy	PS	clinical,culture+	ICU	CD64	93	55	1	6	31	90.1	96.9	58.7	FCM
Ruokonen [52]	2002	Switzerla-nd	PS	clinical,culture+	ICU	PCT	208	110	24	52	22	67.9	47.8		IF
Selberg [53]	2000	Germany	PS	clinical,culture+	ICU	PCT	33	19	5	3	6	86	54	47.9	IF
						IL-6	33	19	5	3	6	86.4	54.5	47.9	EIA
Shokouhi [22] a	2017	Iran	PS	culture+		PCT	192	76	18	16	82	82.6	82	43.9	ELISA
Shokouhi [22] b	2017	Iran	PS	culture+		PCT	184	58	30	26	70	69.1	70	73.1	ELISA
Spoto [54]	2018	Italy	PS	clinical, culture+	ICU/GW	PCT	159	60	1	49	49	55	98	70.5	IF
Suprin [55]	2000	France	PS	clinical. culture+	ICU	PCT	95	49	6	26	14	65.3	70	57	IF
Talebi-Taher [20]	2014	Iran	PS	clinical	ED	PCT	100	44	14	6	36	88.8	71.1	76.3	IF
Tan [56]	2016	Malaysia	PS	clinical,culture+	ED	CD64	51	34	1	8	8	80.9	88.9	53.7	FCM
Tromp [57]	2002	Netherla-nds	PS	culture+	ED	PCT	342	49	120	6	167	89.1	58.2		IF
						IL-6	342	34	79	21	208	61.8	72.5		EI
Tsalik [58]	2012	America	PS	clinical,culture+	ED	PCT	336	168	33	79	56	68	62.9		ECLI
						IL-6	336	144	29	103	60	58.3	67.4		ECLI
Wang [59]	2013	China	RS	culture+	ICU	PCT	586	100	162	20	304	83.3	65.2		IF
Zhang [21]	2017	China	PS	clinical	ICU	PCT	70	36	6	14	14	72	70	92.6	ECLI
Huang [60]	2012	China	PS	clinical	ICU	PCT	72	40	3	9	20	82.3	84.9	66.2	ELISA
Lu [19]	2016	China	PS	clinical	ICU	CD64	420	111	35	19	255	85.1	87.8		FCM
Shao [61]	2014	China	PS	clinical	ICU/RD	CD64	87	63	4	6	14	91.3	77.8	54	FCM
Tang [62]	2017	China	PS	clinical	ICU	PCT	221	74	24	15	108	83.2	82.1	51.6	ECLI
				clinical	ICU	IL-6	221	67	77	22	55	75.3	41.2	51.6	ECLI
Wang [63]	2017	China	PS	clinical	MD	CD64	44	23	1	6	14	79.5	93.3	47.1	FCM
Xing [64]	2008	China	PS	clinical	ED/GW/ICU	PCT	149	84	6	8	51	91.3	89.5	67.3	IF
Xu [65]	2009	China	PS	clinical	ICU/HD	CD64	68	57	1	1	9	98.3	90		FCM
Zhang [66]	2012	China	PS	clinical		CD64	55	30	5	5	15	85.7	75	50.6	FCM
Zhao [67]	2017	China	PS	clinical	ICU	PCT	104	67	5	11	21	85.9	81.8	57.9	IF
Zhao [68]	2016	China	RS	clinical	ED	PCT	393	255	10	52	76	83.2	88.1	42	ECLI
						IL-6	393	249	14	58	72	81.1	83.7	42	EIA
Zhao [69]	2014	China	PS	clinical	ED	PCT	652	340	40	112	160	75.2	80	72	ELISA
						IL-6	652	366	78	86	122	81	61	72	EIA

*PS* Prospective Study, *RS* Retrospective Study, *ICU* intensive care unit, *SICU* Surgical intensive care unit, *MICU* Medical Intensive Care Unit, *RICU* Respiratory intensive care unit, *ED* Emergency Department, *GW* General ward, *RD* Respiratory Department, *HD* Hematology Department, *IF* Immunofluorescence, *FCM* flow cytometer, *ECLI* Electrochemical immunoluminescence, *EIA* enzyme imrrmnoassay, *ELISA* enzyme linked immunosorbent assay

### Quality assessment

**Fig. 2:**
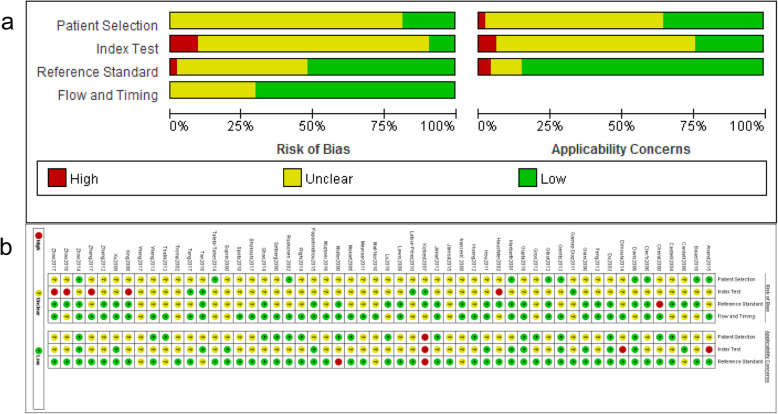
**a** Risk of bias. **b** Clinical applicability

We used the QUADAS-2 scale to evaluate the quality of the included articles. The results showed that all studies were of high quality and had clinical practicability (Fig. 2).

### Heterogeneity test

**Fig. 3:**
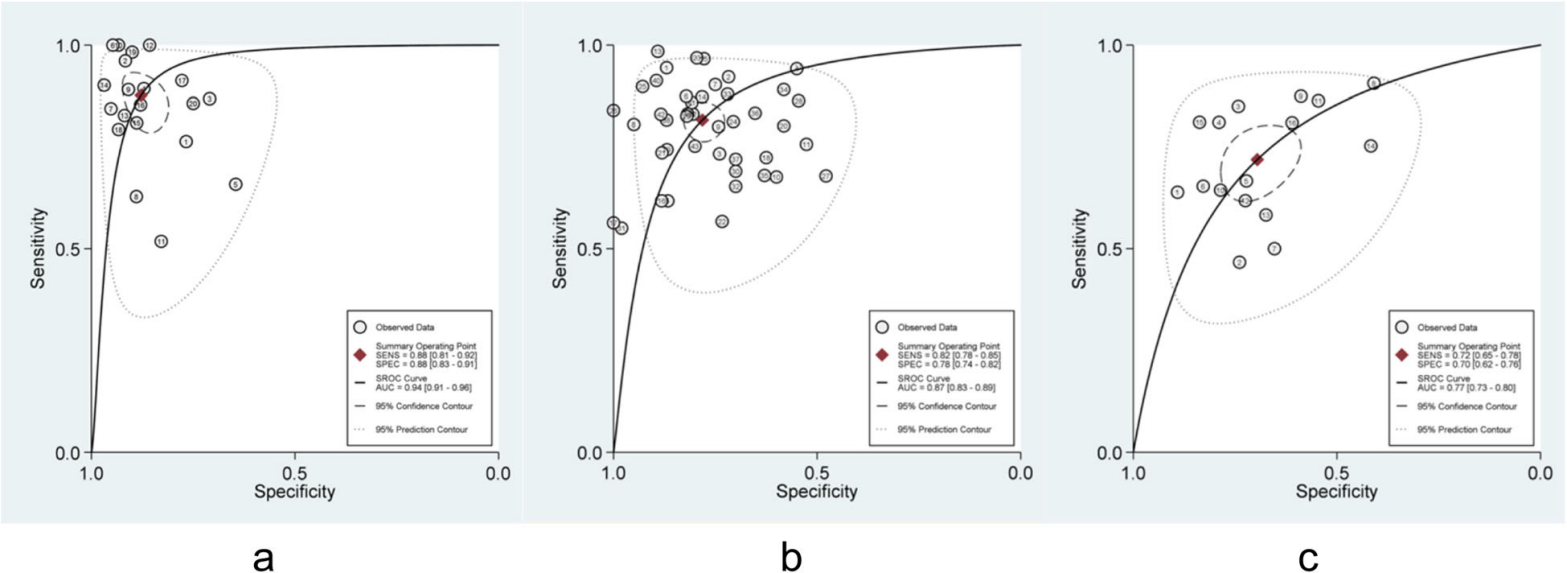
Summary receiver operator characteristic (SROC) of CD64 (**a**) across 20 studies, PCT (**b**) across 43 studies, and IL-6 (**c**) across 16 studies

Spearman correlation coefficients of neutrophil CD64, PCT, and IL-6 were − 0.22 (*P* = 0.35), − 0.054 (*P* = 0.729,) and 0.326 (*P* = 0.217), respectively. The SROC curve of the three biomarkers did not show a significant shoulder-arm effect, suggesting that there was no threshold effect (Fig. 3).

### Pooled effect size result

**Fig. 4:**
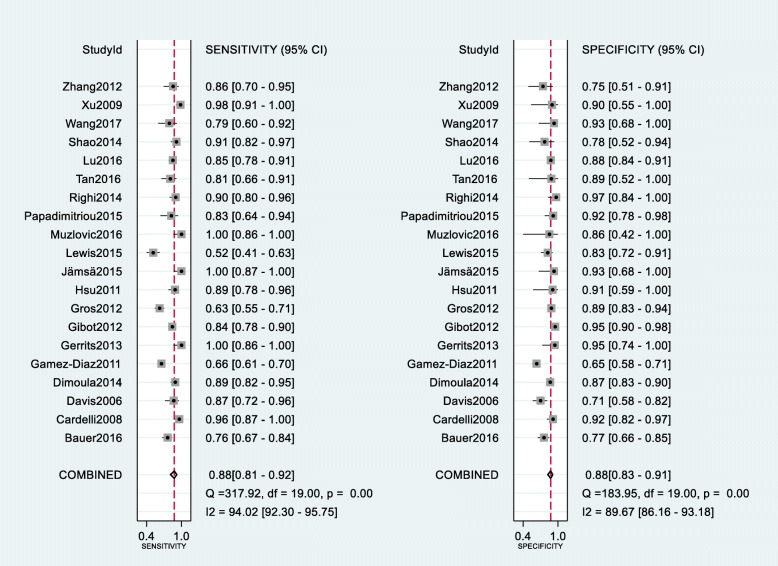
Forest plots of the sensitivity and specificity for CD64 with a 95% confidence interval on the 20 included studies

**Fig. 5:**
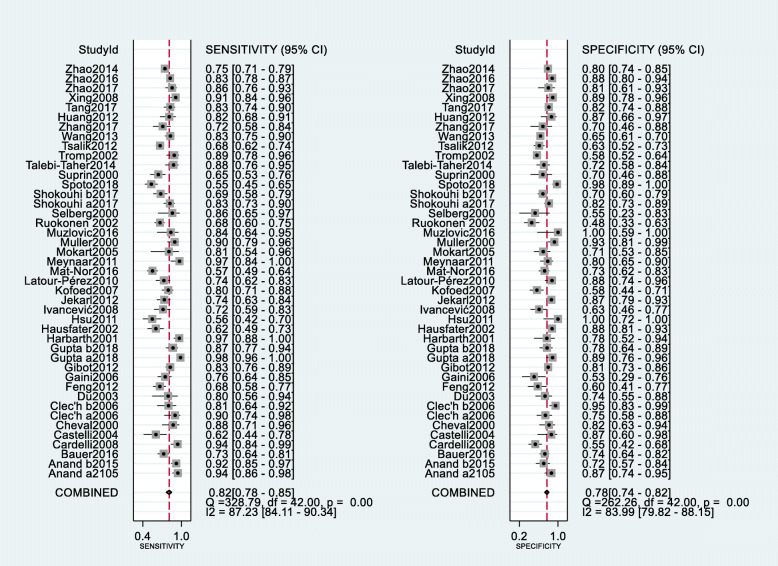
Forest plots of the sensitivity and specificity for PCT with a 95% confidence interval on the 43 included studies

**Fig. 6:**
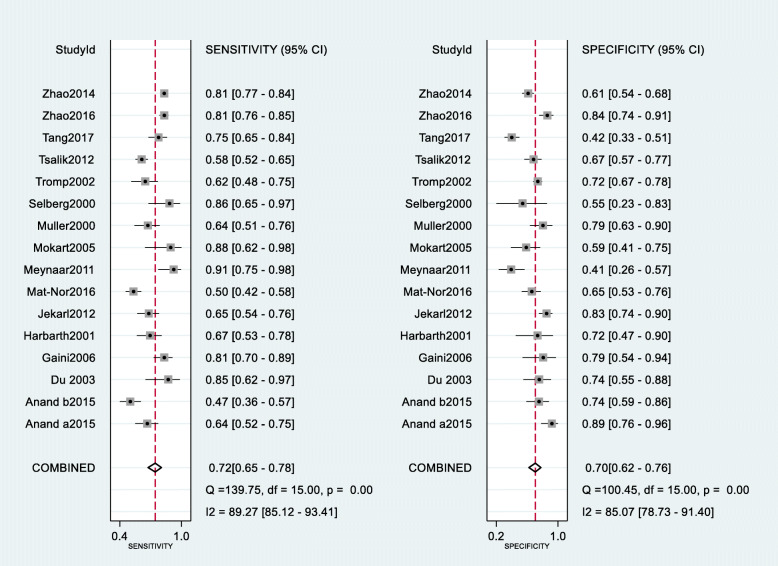
Forest plots of the sensitivity and specificity for IL-6 with a 95% confidence interval on the 16 included studies

Of all included articles, 20 of them reported the diagnostic value of neutrophil CD64 for sepsis. The results for these studies were: pooled sensitivity, 0.88 (95% CI, 0.81–0.92); pooled specificity, 0.88 (95% CI, 0.83–0.91) (Fig. 4); pooled PLR, 7.2 (95% CI, 5.0–10.3); pooled NLR, 0.14 (95% CI, 0.09–0.22); pooled DOR, 51 (95% CI, 25–105); and the AUC was 0.94 (95% CI, 0.91–0.96) (Fig. 3a). Thirty-nine studies reported the diagnostic value of PCT with the following results: pooled sensitivity, 0.82 (95% CI, 0.78–0.85); pooled specificity, 0.78 (95% CI, 0.74–0.82) (Fig. 5); pooled PLR, 3.7(95% CI, 3.1–4.50); pooled NLR, 0.23 (95% CI, 0.19–0.29); pooled DOR, 16 (95% CI, 11–23); and the AUC was 0.87 (95% CI, 0.83–0.89) (Fig. 3b). We found 15 articles reporting the diagnostic value of IL-6 for sepsis. The results for this set of articles were: pooled sensitivity, 0.72 (95% CI, 0.65–0.78); pooled specificity, 0.70 (95% CI, 0.62–0.76) (Fig. 6); pooled PLR, 2.4 (95% CI, 1.9–3.0); pooled NLR, 0.40 (95% CI, 0.32–0.51); pooled DOR, 6 (95% CI, 4.0–9.0); and the AUC was 0.77 (95% CI, 0.73–0.80) (Fig. 3c).

### Publication bias analysis

**Fig. 7:**
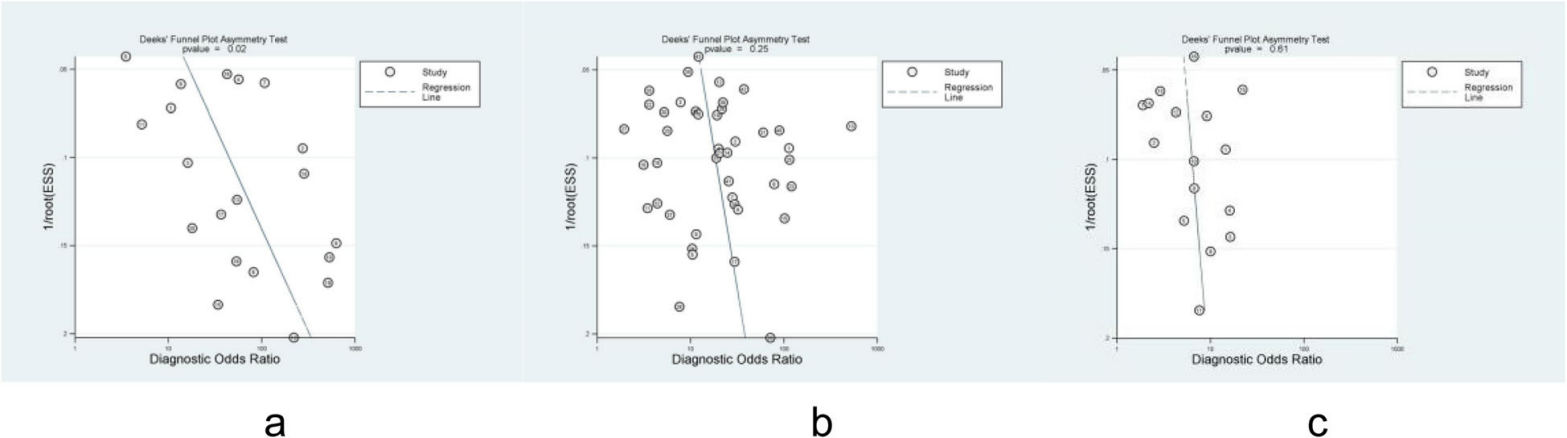
Deeks’ funnel figure for the assessment of potential publication bias for neutrophil CD64 (**a**), PCT (**b**), and IL-6 (**c**) expression in the diagnosis of sepsis

Publication bias of studies regarding neutrophil CD64 showed that 20 articles were not evenly distributed on both sides of the regression line (t = 2.45, *P* = 0.025) (Fig. 7a), suggesting a publication bias among the included studies. No significant bias was found for studies addressing PCT (t = 1.17, *P* = 0.249) (Fig. 7b) or IL-6(t = 0.53, *P* = 0.607) (Fig. 7c).

### Heterogeneity analysis

#### Meta-regression

**Fig. 8:**
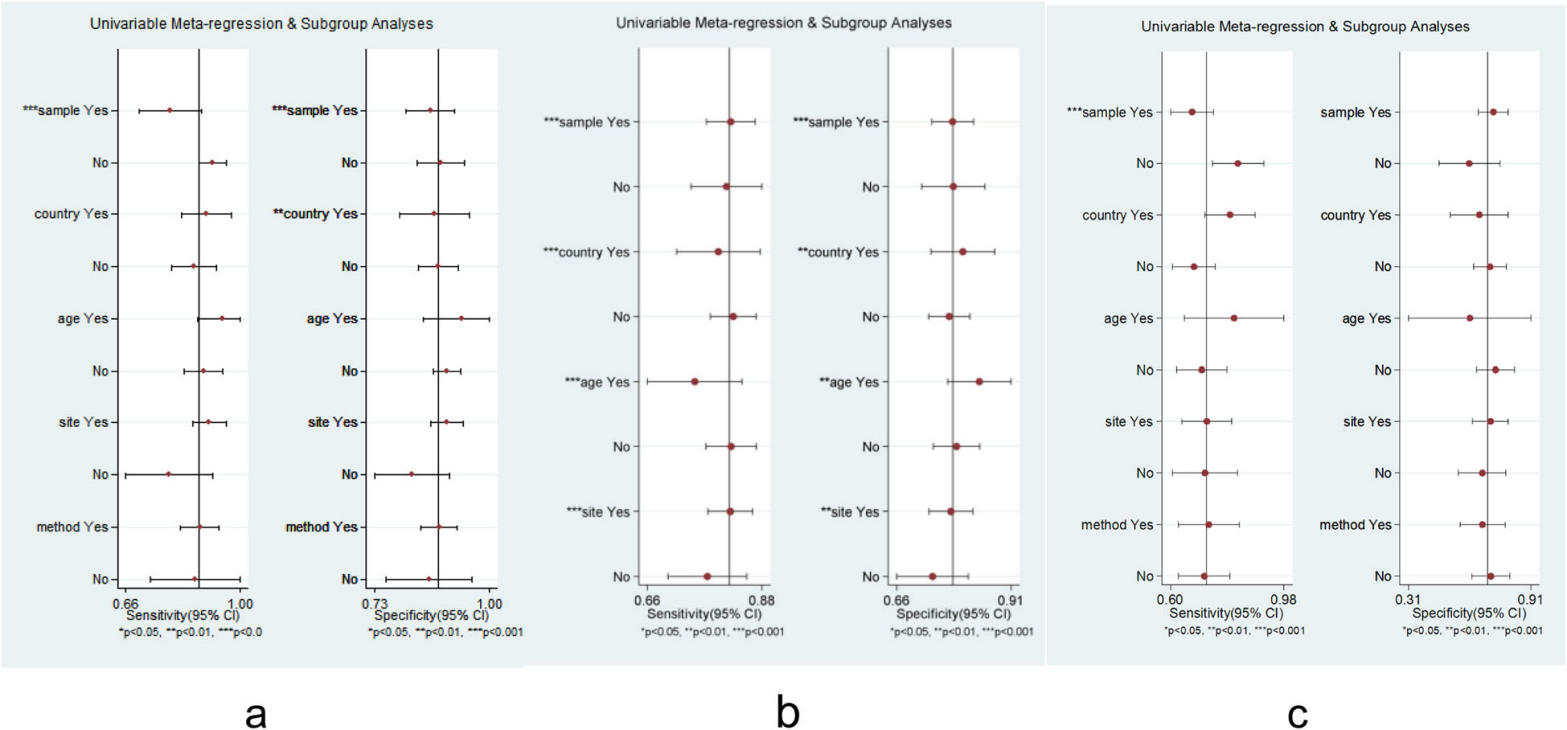
Meta-regression for neutrophil CD64 (**a**), PCT (**b**), and IL-6 (**c**) expression in the diagnosis of sepsis. Meta-regression was performed according to whether the sample size of the study was over 100, study subjects were Chinese, the average age of the study population was over 65, and the clinical setting was classified into ICU and measuring methods

Due to the heterogeneity caused by a non-threshold effect in the included studies, meta-regression was performed when the following criteria were met: a sample size of the study over 100; the patients were Chinese; the average age of patients was over 65 years old; the clinical setting was classified into ICU; and similar test methods were used. The meta-regression of neutrophil CD64 showed that the sample size had an influence on the heterogeneity of sensitivity and specificity, and regional difference was one of the factors that caused the heterogeneity of specificity (Fig. 8a). The meta-regression of PCT showed that the above five factors are likely to be the sources of heterogeneity (Fig. 8b). The meta-regression result of IL-6 indicated that the source of heterogeneity might be the sample size (Fig. 8c).

#### Sensitivity analysis

**Fig. 9:**
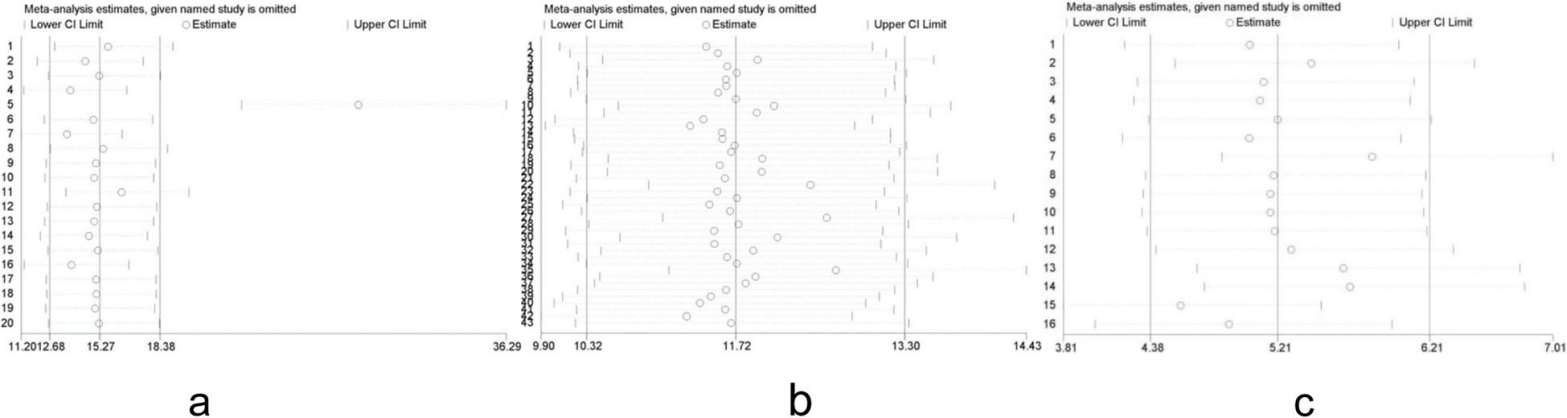
Sensitivity analysis for neutrophil CD64 (**a**), PCT (**b**), and IL-6 (**c**) expression in the diagnosis of sepsis

Concerning the sensitivity analysis of neutrophil CD64, we found that when the article by the Gámez-Díaz et al. [37] study was removed from the subset of studies, the overall heterogeneity of specificity of the 19 articles was decreased, suggesting that the Gámez-Díaz study was the cause for the heterogeneity of specificity (Fig. 9a). When the other 19 studies were removed one by one, the sensitivity, specificity, and AUC showed no significant change. The sensitivity analysis of PCT and IL-6 showed that the sensitivity, specificity, and AUC did not change significantly when they were removed one by one (Fig. 9b, c).

#### Subgroup analysis

Through a sensitivity analysis of neutrophil CD64, it was found that the Gámez-Díaz et al. [37] study had an influence on the heterogeneity of neutrophil CD64, so a subgroup analysis was conducted after excluding such study. The subgroup analysis of three biomarkers indicated that the sample size might be the source of heterogeneity, since the heterogeneity decreased significantly in the group when a small sample size was analyzed, which might be due to the large number of included cases, and a lack of consistency (Tables 2, 3, 4). The subgroup analysis of neutrophil CD64 indicated that regional differences were also a source of heterogeneity, which was consistent with the meta-regression results. Heterogeneity decreased significantly in the Chinese group but remained high in the non-Chinese group. The subgroup analysis showed that the sensitivity, specificity, and AUC of neutrophil CD64 in non-elderly patients were 0.89 (95% CI, 0.91–0.94), 0.90 (95% CI, 0.86–0.93), 0.94 (95% CI, 0.91–0.96), respectively. The sensitivity, specificity, and AUC of PCT in ICU patients were 0.82 (95% CI, 0.77–0.86), 0.78 (95% CI, 0.72–0.82), 0.86 (95% CI, 0.83–0.89), respectively; the SEN, SPE, and AUC of PCT in non-ICU patients were 0.77 (95% CI, 0.72–0.82), 0.74 (95% CI, 0.64–0.81), and 0.82 (95% CI, 0.78–0.85), respectively.

**Table 2:** Subgroup analysis of CD64 in the diagnosis of sepsis

category	studies	SEN (95% CI)	SPE (95%CI)	DOR (95% CI)	AUC (95% CI)	SEN-I^2^ (%)	SPE-I^2^ (%)
overall	19	0.89 [0.82, 0.93]	0.88 [0.84,0.92]	59 [30, 115]	0.94 [0.91,0.96]	90.39	76.03
subgroup analysis based on sample size							
size≥100	8	0.82 [0.71,0.89]	0.87 [0.81,0.91]	29 [13,64]	0.91 [0.88,0.93]	91.53	78.72
size< 100	11	0.92 [0.86,0.96]	0.90 [0.84,0.94]	105 [44,252]	0.95 [0.93,0.97]	62.09	13.49
subgroup analysis based on country							
China	6	0.89 [0.84, 0.93]	0.86 [0.80,0.91]	53 [30, 92]	0.92 [0.89, 0.94]	49.79	0.00
non-China	13	0.88 [0.79, 0.94]	0.89 [0.84,0.93]	64 [24, 168]	0.94 [0.92, 0.96]	92.42	83.07
subgroup analysis based on patient scource							
ICU	13	0.89 [0.80, 0.94]	0.90 [0.86,0.93]	73 [29, 183]	0.94 [0.92, 0.96]	93.18	78.96
non-ICU	4	–	–	–	–	–	–
subgroup analysis based on assay method							
FMC	16	0.87 [0.82, 0.91]	0.88 [0.83,0.91]	50 [27, 96]	0.94 [0.91, 0.96]	86.71	71.13
Leuko64 kit	3	–	–	–	–	–	–
subgroup analysis based on mean age							
age ≥ 65 y	2	–	–	–	–	–	–
age < 65 y	11	0.89 [0.81, 0.94]	0.90 [0.86,0.93]	77 [37, 164]	0.94 [0.91, 0.96]	90.02	61.12

**Table 3:** Subgroup analysis of PCT in the diagnosis of sepsis

category	studies	SEN (95% CI)	SPE (95%CI)	DOR (95% CI)	AUC (95% CI)	SEN-I^2^ (%)	SPE-I^2^ (%)
overall	43	0.82[0.78, 0.85]	0.78[0.74,0.82]	16[11, 23]	0.87[0.83,0.89]	87.23	83.99
subgroup analysis based on sample size							
size≥100	27	0.82[0.77,0.86]	0.78[0.73,0.83]	16[11,25]	0.87[0.84,0.90]	90.42	88.98
size< 100	16	0.81[0.74,0.86]	0.78[0.71,0.83]	15[9.25]	0.86[0.83,0.89]	74.74	52.18
subgroup analysis based on country							
China	11	0.79[0.74, 0.84]	0.79[0.73,0.85]	15[8, 26]	0.86[0.83, 0.89]	78.26	83.92
non-China	33	0.83[0.77, 0.87]	0.77[0.72,0.82]	16[11, 25]	0.87[0.84, 0.89]	89.29	84.48
subgroup analysis based on patient scource							
ICU	27	0.82[0.77, 0.86]	0.78[0.72,0.82]	16[10, 24]	0.86[0.83, 0.89]	86.20	76.10
non-ICU	10	0.77[0.72, 0.82]	0.74[0.64,0.81]	9[6, 15]	0.82[0.78, 0.85]	74.39	90.16
subgroup analysis based on mean age							
age ≥ 65 y	8	0.79[0.72, 0.8]	0.84[0.75,0.90]	20[12, 34]	0.88[0.85, 0.91]	86.45	74.39
age < 65 y	20	0.80[0.73, 0.86]	0.81[0.76,0.85]	17[10, 29]	0.87[0.84, 0.90]	84.01	73.73

**Table 4:** Subgroup analysis of IL-6 in the diagnosis of sepsis

category	studies	SEN (95% CI)	SPE (95%CI)	DOR (95% CI)	AUC (95% CI)	SEN-I^2^ (%)	SPE-I^2^ (%)
overall	16	0.72[0.65, 0.78]	0.70[0.62,0.76]	6[4, 9]	0.77[0.73,0.80]	89.27	85.07
subgroup analysis based on sample size							
size≥100	10	0.66[0.58,0.3]	0.73[0.64,0.80]	5[3,8]	0.75[0.71,0.78]	92.34	88.99
size< 100	6	0.83[0.73,0.83]	0.64[0.51,0.75]	8[5,14]	0.81[0.77,0.84]	52.42	62.91
subgroup analysis based on country							
China	4	–	–	–	–	–	–
non-China	12	0.69[0.59, 0.77]	0.70[0.63,0.77]	5[3, 8]	0.75[0.71, 0.79]	80.86	74.47
subgroup analysis based on patient scource							
ICU	10	0.71[0.60, 0.80]	0.74[0.66,0.81]	8[4, 14]	0.80[0.76, 0.83]	91.94	80.76
non-ICU	6	0.73[0.64, 0.80]	0.66[0.54,0.75]	5[3, 8]	0.74[0.70, 0.78]	84.28	84.97
subgroup analysis based on assay method							
EIA	8	0.75[0.64, 0.83]	0.70[0.63,0.76]	7[4, 12]	0.77[0.73, 0.81]	91.31	67.89
ECLI	8	0.69[0.59, 0.77]	0.69[0.56,0.80]	5[3, 9]	0.75[0.71, 0.78]	83.28	90.73
subgroup analysis based on mean age							
age ≥ 65 y	1	–	–	–	–	–	–
age < 65 y	9	0.71[0.61, 0.79]	0.74[0.63, 0.82]	7[4, 13]	0.78 [0.75,0.82]	90.46	90.59

#### Clinical utility evaluation

**Fig. 10:**
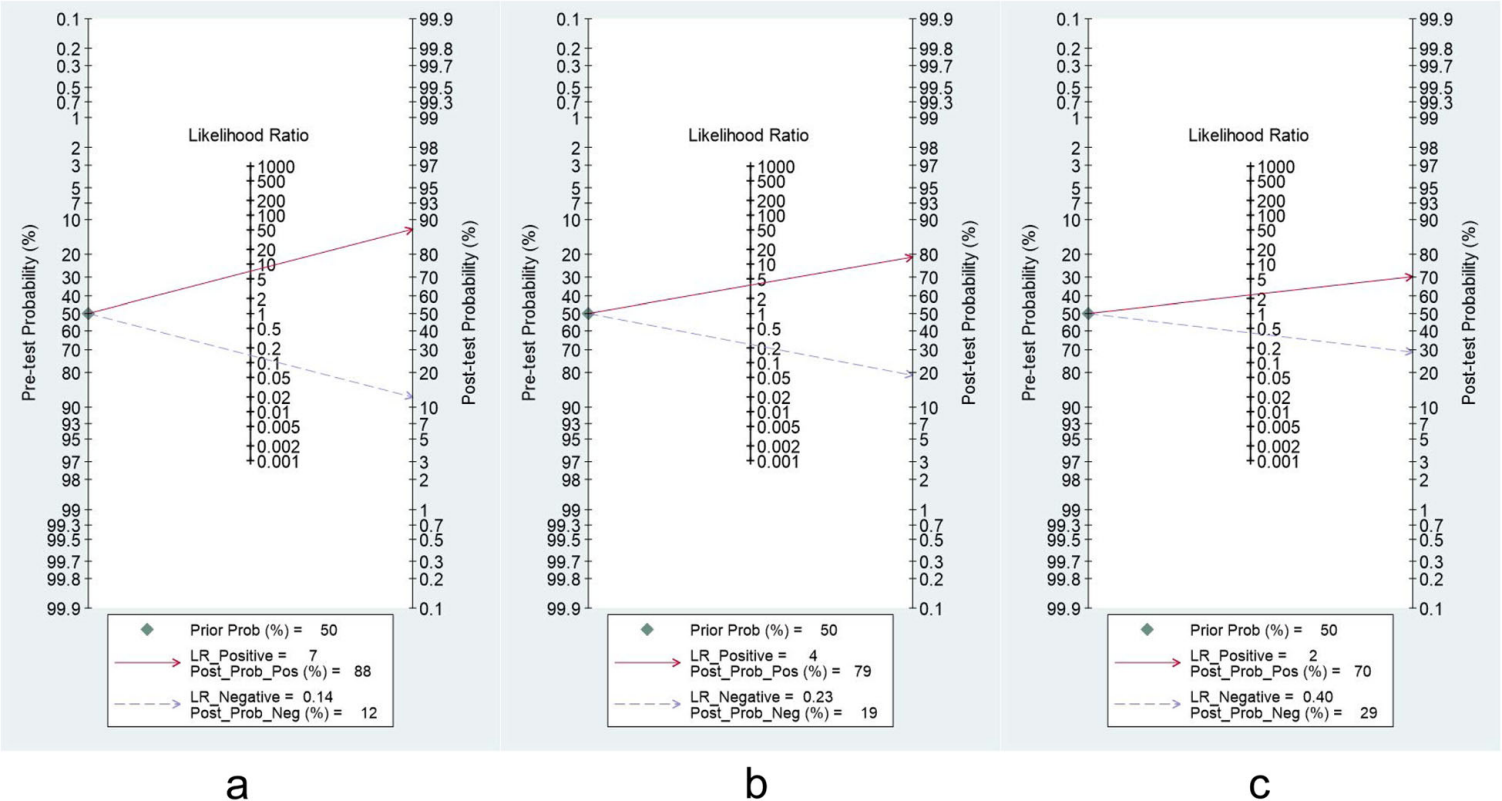
Fagan’s nomogram for neutrophil CD64 (**a**), PCT (**b**), and IL-6 (**c**) expression in the diagnosis of sepsis

We assumed a pre-test probability of 50%. The Fagan’s nomogram of neutrophil CD64 showed a post-test probability of 88% positive and 12% negative (Fig. 10a). The Fagan’s nomogram of PCT showed a post-test probability of 79% positive and of 19% negative (Fig. 10b), whereas the Fagan’s nomogram of IL-6 showed a post-test probability of 70% positive and of 29% negative (Fig. 10c).

## Discussion

Our results showed that neutrophil CD64 had the highest diagnostic value for sepsis in adult patients with a pooled sensitivity of 0.88 (95% CI, 0.81–0.92); pooled specificity of 0.88 (95% CI, 0.83–0.91); and AUC of 0.94 (95% CI, 0.91–0.96), followed by PCT, with a pooled sensitivity of 0.82 (95% CI, 0.78–0.85); pooled specificity of 0.78 (95% CI, 0.74–0.82); and AUC of 0.87 (95% CI, 0.83–0.89). Of all three studied biomarkers, IL-6 showed the weakest diagnostic value for sepsis, with a pooled sensitivity of 0.72 (95% CI, 0.65–0.78), the pooled specificity of 0.70 (95% CI, 0.62–0.76), and AUC of 0.77 (95% CI, 0.73–0.80).

In 2006, Davis et al. [30] reported for the first time the diagnostic potential of neutrophil CD64 in sepsis patients through a retrospective review of 100 blood samples and showed that the performance of neutrophil CD64 was better than white blood cell count, erythrocyte sedimentation, and C-reactive protein as a sepsis diagnostic marker. In the past 10 years, some prospective studies have shown the clinical value of CD64 in the diagnosis of sepsis. In previous studies, Hsu et al. [41] found that the accuracy of neutrophil CD64 was better than PCT in respiratory intensive care unit patients to distinguish systemic inflammatory response syndrome from severe sepsis and septic shock. Neutrophil CD64 was also found to be associated with mortality. However, some studies criticized the diagnostic value of neutrophil CD64 in sepsis. Gros et al. [38] showed that neutrophil CD64 has a low sensitivity in the diagnosis of sepsis in ICU or emergency department patients. However, due to its high specificity, when combined with other sensitive markers, it may contribute to the clinical diagnosis of sepsis. In 2016, Wang et al. [70] conducted a meta-analysis with 8 studies written in English, to assess the value of neutrophil CD64 for the diagnosis of sepsis. The results showed that the pooled sensitivity, specificity, and AUC were 0.76, 0.85, and 0.95 respectively, which suggested that neutrophil CD64 had a high specificity for sepsis. However, because of its low sensitivity, it could not be used alone in the diagnosis of sepsis. Our meta-analysis searched publications in more databases than other published meta-analysis, more comprehensive clinical research data was collected, and the results were more persuasive. In our study, 20 studies were included, showing that the neutrophil CD64 test has a high sensitivity and specificity in adult sepsis patients, and was superior to the traditional biomarkers PCT and IL-6. Li et al. [71] carried out a meta-analysis to evaluate the diagnostic value of CD64 in infectious diseases, including adults and newborns. The results showed that the pooled sensitivity, specificity, and AUC were 0.76, 0.85, and 0.92 respectively, which suggested that the neutrophil CD64 had a high specificity in sepsis. Due to the uniqueness of neonate sepsis in many aspects, our study only included studies on adult sepsis patients.

Although IL-6 is weaker than the neutrophil CD64 and PCT in the diagnosis of sepsis in adult patients, some studies have shown that it also plays a role in the prognosis of infectious diseases [72, 73]. Studies have found that the level of IL-6 in the blood of patients with Gram-negative bacterial infection is significantly higher than those with Gram-positive bacterial infection [74], indicating that IL-6 has a certain suggestive effect on the pathogenic bacteria. Zhao et al. [75] through the regression analysis results show that a combination of the three biomarkers (PCT, IL-6, and D-dimer) can effectively improve the diagnosis of sepsis and severe sepsis. However, joint diagnosis in clinical research data is uncommon and there is not enough to apply to meta-analysis for data integration to further explore this topic.

We used sensitivity analysis, meta-regression, and subgroup analysis to explore the heterogeneity of data. The sensitivity analysis showed that the heterogeneity decreased significantly when the Gámez-Díaz et al. [35] study was omitted. The sample size of this study was the largest among all included studies, and the study results were negative, which could lead to an increase in heterogeneity. The meta-regression and subgroup analysis indicated several factors can explain the heterogeneity that we observed, including regional difference, differently aged patients, the sample size, the severity of the disease, and test methods. Through the subgroup analysis of the articles, we found that the specificity of the neutrophil CD64 in non-elderly patients has increased compared to all ages. Further studies to determine the accuracy of neutrophil CD64 in differently aged patients are required. PCT in the ICU group has a higher diagnostic efficacy for sepsis than in the non-ICU group. The study of Yunus et al. [76] found PCT was positively correlated with the severity of sepsis. Because the proportion of patients with severe sepsis and septic shock among ICU patients was large, the PCT in the ICU patients showed a better diagnostic efficiency. PCT had a better diagnostic value in critically ill patients than in those with non-severe conditions.

Our research is limited by some factors. Firstly, the heterogeneity in the study is high. Although some sources of heterogeneity have been found through meta-regression, sensitivity analysis, and subgroup analysis, there are still other unidentified sources. Secondly, there is a publication bias in the analysis of the diagnostic accuracy of sepsis toward neutrophil CD64. In the follow-up of this study, the scope should be expanded to overcome the publication bias. Thirdly, only Chinese and English language literature was included, which might exclude relevant data. Fourthly, due to the different test methods for the three biomarkers, the cut-off values varied between the included studies. Future studies are needed to determine the optimal cut-off value of biomarkers that confers the diagnostic value for sepsis.

## Conclusions

Among the three biomarkers, neutrophil CD64 has the highest diagnostic value for sepsis in adult patients, followed by PCT and IL-6. In the diagnosis of sepsis, the diagnostic value of PCT in severe patients is better than that in non-severe patients.

## Data Availability

All relevant data supporting the conclusion of this study are included within the paper.

## Abbreviations

AUC: Area under the curve
DOR: Diagnostic odds ratio
FN: False negative
FP: False positive
ICU: Intensive care unit
IL-6: Interleukin-6
LPS: Lipopolysaccharides
NLR: Negative likelihood ratio
PCT: Procalcitonin
PLR: Positive likelihood ratio
QUADAS: Quality Assessment for Diagnostic Accuracy Studies
SEN: Sensitivity
SOFA: Sequential Organ Failure Assessment
SPE: Specificity
SROC: Summary Receiver Operating Characteristic
TN: True negative
TP: True positive
